# Hydrogen Sulfide: A Potent Tool in Postharvest Fruit Biology and Possible Mechanism of Action

**DOI:** 10.3389/fpls.2018.01375

**Published:** 2018-09-19

**Authors:** Vasileios Ziogas, Athanassios Molassiotis, Vasileios Fotopoulos, Georgia Tanou

**Affiliations:** ^1^Institute of Olive Tree, Subtropical Plants and Viticulture, ELGO-DEMETER, Chania, Greece; ^2^Laboratory of Pomology, Department of Agriculture, Aristotle University of Thessaloniki, Thessaloniki, Greece; ^3^Department of Agricultural Sciences, Biotechnology and Food Science, Cyprus University of Technology, Limassol, Cyprus; ^4^Institute of Soil and Water Resources, ELGO-DEMETER, Thessaloniki, Greece

**Keywords:** ethylene, fruit ripening, hydrogen sulfide, postharvest biology, reactive nitrogen and oxygen species, *S-*sulfhydration

## Abstract

Hydrogen sulfide (H_2_S), an endogenous gaseous molecule, is considered as a signaling agent, in parallel with other low molecular weight reactive substances, mainly hydrogen peroxide (H_2_O_2_) and nitric oxide (NO), in various plant systems. New studies are now revealing that the postharvest application of H_2_S, through H_2_S donors such as sodium hydrosulfide (NaSH) or sodium sulfide (Na_2_S), can inhibit fruit ripening and senescence programs in numerous fruits. We discuss here current knowledge on the impact of H_2_S in postharvest physiology of several climacteric and non-climacteric fruits such as banana, apple, pear, kiwifruit, strawberry, mulberry fruit, and grape. Although there is still a considerable lack of studies establishing the mechanisms by which H_2_S signaling is linked to fruit metabolism, we highlight several candidate mechanisms, including a putative cross-talk between H_2_S and ethylene, reactive oxygen and nitrogen species, oxidative/nitrosative stress signaling, sulfate metabolism, and post-translational modification of protein cysteine residues (*S-*sulfhydration) as being functional in this H_2_S postharvest action. Understanding H_2_S metabolism and signaling during postharvest storage and the interplay with other key player molecules would therefore provide new, improved strategies for better fruit postharvest storage. To achieve this understanding, postharvest fruit physiology research will need to focus increasingly on the spatial interaction between H_2_S and ethylene perception as well as on the interplay between *S*-sulfhydration/desulfhydration and *S*-nitrosylation/denitrosylation under several postharvest conditions.

## Introduction

The continuously increasing world population demands more effective food production strategies, better agriculture management systems, and less postharvest losses (Kader, 2005). Undertaken research by various international and national organizations led by FAO indicated that about one-third of all food produced on the planet and about a half of all fruit and vegetables are lost and not consumed (Porat et al., 2018). Postharvest attributes of horticultural products are associated with the use of synthetic chemicals; however, the application of chemicals as germicides raises several issues related to pathogen resistance and food safety (Deng et al., 2013). Therefore, research has set as a priority the establishment of consumer friendly postharvest treatments (Gong et al., 2018). Toward this goal, several low molecular weight compounds, such as hydrogen sulfide (H_2_S), nitric oxide (NO), hydrogen peroxide (H_2_O_2_), hydrogen gas (H_2_), carbon dioxide (CO_2_), and chlorine dioxide (ClO_2_) have been applied to perishable horticultural products (Gong et al., 2018). Herein, we focus upon the latest research data linked with the physiological aspect of H_2_S application in the fruit postharvest behavior along with future perspectives.

### Physiology, Biochemistry, and Signaling Action of H_2_S in Plants: A Brief Account

Hydrogen sulfide research began upon animal systems, but during the last decade, a plethora of scientific data originated from plants (Wang, 2002). A significant body of evidence suggest that at minor concentrations H_2_S can exert signaling properties during the acclimation of plants to abiotic stress, plant growth and development, and specific physiological processes through an interplay with hormones, reactive oxygen species (ROS), and other signaling compounds, like NO (Hancock et al., 2011; Christou et al., 2014; Hancock and Whiteman, 2014, 2016; Ziogas et al., 2015; Antoniou et al., 2016).

In plants, H_2_S is produced through sulfite reductase, which catalyzes the reduction of sulfite to sulfide, or through two cysteine-dependent reactions involving members of the *O*-acetylserine(thiol)lyase (OAS-TL) gene family. L-cysteine desulfhydrase (DES, EC 4.4.1.1) converts L-cysteine to H_2_S, ammonia, and pyruvate while β-cyanoalanine synthase produces H_2_S through the detoxification of cyanide at the expense of cysteine (Hatzfeld et al., 2000). Although the biochemical aspects of H_2_S are extended, it has been suggested that H_2_S exerts its biological activity mainly *via* the oxidative post-translational modification of cysteine residues (RSH) to persulfides (RSSH; Filipovic and Jovanović, 2017).

## The Emerging Role of H_2_S in Postharvest Physiology of Fruits

Fruit ripening is accompanied by various biochemical and physiological changes, which are orchestrated by multiple genetically programmed processes (Gong et al., 2018). Based on the physiological differences in respiratory pattern during ripening, fleshy fruits have been categorized as climacteric and non-climacteric. Fleshy fruits have long been categorized to climacteric or non-climacteric according to various biochemical differences of their respiratory pattern during ripening. A characteristic burst of ethylene production has been observed to climacteric fruits like banana, apple, and kiwifruit, while non-climacteric products, like strawberry and grape, withhold ethylene production at basal level (Cherian et al., 2014). Climacteric fruits such as banana, apple, and kiwi display a well-characterized peak in respiration with a concomitant burst of ethylene at the onset of ripening. In contrast, non-climacteric fruits, which include strawberry and grape, do not show a dramatic change in respiration, and ethylene production remains at a basal level (Cherian et al., 2014). During the last decade, research has focused on the role of low molecular compounds that manipulate metabolic pathways linked with freshness and extended postharvest life of horticultural produce (Gong et al., 2018). Although the potential mechanism is poorly understood (Fotopoulos et al., 2015), increasing evidence suggest that H_2_S significantly influences the postharvest life of fruits from perennial plants (**Table 1**).

**Table 1:** Different effects of H_2_S on postharvest life of fruits from perennial plants.

Plant species	Treatment	Physiological outcome	Reference
Strawberry (*Fragaria ananassa* L. “Fengxiang”)	NaHS + sodium nitroprusside (SNP; a NO donor)	Suppress fruit decay	Zhang et al., 2014
		Inhibit respiration rate	
		Maintain crust color	
		Preserve fruit quality (firmness-relative conductivity)	
		CHI↑, GNS↑, PME↓, PG↓, EGase↓	
Strawberry (*Fragaria* x *ananassa* Duch., cv. Bao Jiao)	NaHS	Higher levels of reducing sugars, soluble proteins, free amino acids, and endogenous H_2_S	Hu et al., 2012
		Lower rot index and respiration rate	
		Higher fruit firmness	
		Reduced ROS and MDA accumulation	
		APX↑, CAT↑, POD↑, GR↑, PG↓, LOX↓	
Kiwifruit (*Actinidia deliciosa*)	NaHS	Higher levels of reducing sugars and soluble proteins, free amino acids, ascorbate, and chlorophyll	Gao et al., 2013
		Reduced levels of carotenoids	
		Reduced ROS and MDA accumulation	
		APX↑, CAT↑, POD↑, GR↑, LOX↓	
Kiwifruit (*Actinidia chinensis* Planch. cv. Jinkui)	NaHS	Inhibit increase in soluble sugars and ethylene production	Zhu et al., 2014
		Maintain higher levels of TA and Vit C	
		Preserve fruit quality (firmness-chlorophyll content)	
		Reduce ROS accumulation	
		CAT↑, POD↑, SOD↑	
Banana (*Musa* spp. AAA group cv. “Brazil”)	NaHS	High levels of lightness, peel firmness, total phenolics, and proline	Luo et al., 2015
		Reduce ROS and MDA accumulation	
		APX↑, CAT↑, POD↑, SOD↑, PAL↑, GR↑, P5CS↑, PDH↓	
Banana (*Musa* spp. AAA group cv. “Brazil”)	NaHS	High levels of peel firmness, hue angle	Li et al., 2016
		Low levels of electrolyte leakage, MDA, and ethylene	
		Enhanced energy metabolism (H^+^-ATPase↑, Ca^2+^-ATPase↑, CCO↑, SDH↑)	
Banana (*Musa* spp. AAA cv. “Brazil”)	NaHS + ethylene	Maintain chlorophyll levels, phenolics	Ge et al., 2017
		Increase flavonoids	
		Decrease carotenoids and soluble sugars in peel	
		Decrease reducing sugars in pulp	
		Reduce ROS and MDA accumulation	
		Increase total antioxidant capacity	
		*MaACS1*↓, *MaACS2*↓, *MaACO1*↓, *MaPL*↓, *MaETR*↑, *MaERS1*↑, *MaERS2*↑	
Mulberry fruit (*Morus indica* L. Dianmian-1)	NaHS	Enhance endogenous H_2_S content	Hu H. et al., 2014
		Delay ripening	
		Reduce respiratory intensity and anthocyanin content	
		Preserve soluble proteins, TA, Vit C	
		Reduce ROS accumulation	
		SOD↑, CAT↑, POD↑, LCD↑, DCD↑	
Apple (*Malus* x *pumila* cv “Fuji”)	NaHS	Preserve Vit C, flavonoids, total phenols, reducing sugars, soluble proteins	Zheng et al., 2016
		Reduce ROS and MDA accumulation	
		APX↑, CAT↑, POD↑, GR↑, SOD↑, LOX↓, PPO↓, PAL↓	
		*MdDHAR*↑, *MdLOX2*↓, *MdPG1*↓, *MdPPO*↓, *MdACO1*↓, *MdERS1*↓, *MdETR1*↓	
Grape (*Vitis vinifera* L. x *V. labrusca* L. Kyoho)	NaHS	Preserve grape cluster weight loss	Ni et al., 2016
		High pulp firmness, soluble solids, TA, Vit C, phenolics, flavonoids, reducing sugars, and soluble proteins	
		Preserve chlorophyll and carotenoid content	
		Reduce ROS and MDA accumulation	
		APX↑, CAT↑, LOX↓	
Pear (*Pyrus pyrifolia*)	NaHS	High levels of reducing sugars and soluble proteins	Hu K.D. et al., 2014
		Reduce ROS and MDA accumulation	
		APX↑, CAT↑, POD↑, LOX↓, PAL↓PPO↓	
		Inhibit fungal growth of *Aspergillus niger* and *Penicillium expansum*	
Apple (*Malus domestica*) Kiwifruit (*Actinidia deliciosa*), Pear (*Pyrus bretschneideri* Rehd.) Sweet Orange (*Citrus sinensis*) Mandarin (*Citrus reticulata*)	NaHS	Inhibit fungal growth of *Aspergillus niger* and *Penicillium italicum*	Fu et al., 2014


### H_2_S Action in Postharvest Physiology: The Climacteric Fruit Model

#### Banana

Banana (*Musa acuminata*) is a typical climacteric fruit, whose ripening initiation is characterized by a sudden increase of ethylene production. Ripe banana fruit suffers from extensive postharvest losses due to ethylene-associated texture softening, peel deterioration, and disease vulnerability (Liu et al., 1999). 1-methylcyclopropene (1-MCP) is a well-known blocker of membrane ethylene receptors, resulting to an extensive shelf life of fruits (Harris et al., 2000). However, the application of 1-MCP upon banana fruits is associated with the negative outcome of green color preservation or yellow color uneven allocation, resulting to limited commercial potential of 1-MCP in banana fruits, thus pinpointing the need to develop alternative mechanism that could manipulate the role of ethylene during ripening (Golding et al., 1998). Ge et al. (2017) showed that the coupled treatment of H_2_S with ethylene in banana fruits downregulated the expression profile of ethylene biosynthesis genes *MaACS1, MaACS2*, and *MaACO1* and pectate lyase *MaPL*, while enhancing the expression profile of ethylene receptor genes *MaETR, MaERS1*, and *MaERS2*, compared with solo ethylene application. In addition, H_2_S treatment sustained fruit chlorophyll content, increased carotenoids, soluble proteins, and the overall antioxidant capacity (Ge et al., 2017). These results suggest that H_2_S delayed banana fruit ripening and senescence via an antagonizing effect with ethylene, through the alleviation of oxidative stress and inhibition of ethylene signaling (Ge et al., 2017).

Cold storage is widely used for the extent of postharvest life of many horticultural products (Wang, 1990). However, due to its tropical nature, banana fruit is prone to chilling injury (CI), which expressed as surface browning, pitting and inability to soften below 13°C (Jiang et al., 2004). Fumigation treatment with H_2_S depressed the development of CI in banana fruit during cold storage under various ripening stages (Luo et al., 2015). In this work, H_2_S also sustained peel firmness and reduced MDA content while phenylalanine ammonia lyase (PAL) activity and antioxidant capacity was increased with parallel increase in the activity of antioxidant enzymes, including superoxide dismutase (SOD), catalase (CAT), ascorbate peroxidase (APX), and glutathione reductase (GR). Finally, H_2_S fumigation increased proline content by promoting the activity of proline biosynthetic enzyme P5CS and suppressing the activity of proline dehydrogenase. A similar study by Li et al. (2016) indicated that banana fruits fumigated with H_2_S under chilling temperature conditions showed higher firmness and lower electrolyte leakage, MDA content, and ethylene production, again indicating a positive effect of H_2_S toward CI in banana fruit. Interestingly, H_2_S treatment enhanced the enzyme activity of H^+^-ATPase, Ca^+2^-ATPase, cytochrome C oxidase (CCO), and succinate dehydrogenase (SDH), highlighting a direct effect on energy metabolism and maintenance of energy charge (Li et al., 2016).

#### Apple

In recent years, there has been a rapid expansion in the sale of fresh-cut apples due to the advantages offered by ready-to-eat or ready-to-use fresh produce (Rico et al., 2007). However, exposure of cut fruit surfaces to atmospheric factors limits their potential for longer postharvest life when compared with intact fruits. The multifunctional signaling role of H_2_S in fresh-cut apples was established by Zheng et al. (2016). Treatment with H_2_S retarded postharvest spoilage of fresh-cut “Fuji” apples (*Malus x pumila*) *via* the modulation of the antioxidant metabolism and the regulation of senescence-related gene expression (Zheng et al., 2016). In detail, H_2_S treatment upon fresh-cut apples retained quality traits like ascorbic acid, flavonoids, total phenolics, reducing sugars, and soluble proteins. Molecular analysis revealed that the delayed postharvest senescence of apple fruits caused by H_2_S was linked with the suppression of genes involved in ethylene biosynthesis (*MdACS1, MdACS3, MdACO1*, and *MdACO2*) and signal transduction (*MdETR1, MdERS1, MdERS2, MdERF3, MdERF4*, and *MdERF5*; Zheng et al., 2016), thereby supporting the counteractive role of H_2_S in ethylene biosynthesis and signaling.

#### Pear

The positive effect of H_2_S treatment (applied as the H_2_S donor NaHS at 0.5–2.5 mM that could liberate about 0.05–0.5 ppm H_2_S gas into a closed container) on fresh-cut pear slices was also reported (Hu K.D. et al., 2014). Particularly, H_2_S fumigation on sliced pears (*Pyrus pyrifolia* cv. Dangshan) caused the maintenance of higher levels of reduced sugars and soluble proteins while reducing the accumulation of ROS and MDA in a dose-dependent manner. These findings further supported the role of H_2_S in antioxidant mechanism, since H_2_S fumigation upregulated the enzymatic activities of APX, CAT, and POD while reduced the enzymatic activities of polyphenol oxidase (PPO), lipoxygenase (LOX), and PAL. Interestingly, postharvest storage of pear slices was also prolonged by the inhibition of fungal pathogens (*Aspergillus niger* and *Penicillium expansum*) due to H_2_S fumigation (Hu K.D. et al., 2014), highlighting its importance as a potent postharvest fungicide agent.

#### Kiwifruit

Kiwifruit is a typical climacteric fruit, and its ripening is closely associated with ethylene biosynthesis. Harvested kiwifruit undergoes a rapid increase of ethylene production after storage, leading to a limited postharvest life at room temperature (Minas et al., 2012; Minas et al., 2014; Tanou et al., 2015; Ainalidou et al., 2016). There is solid evidence that H_2_S delayed kiwifruit (*Actinidia chinensis* Planch. cv. Jinkui) postharvest ripening, expressed as titratable acidity (TA) and ethylene production. Treatment with H_2_S also increased the activity of SOD, POD, and CAT resulting in direct decrease of accumulated ROS, ultimately protecting kiwifruit cell membranes during postharvest storage (Zhu et al., 2014). The previously presented diverse positive effects of H_2_S on kiwifruit postharvest behavior were supported by another report pinpointing that fumigation of fresh-cut kiwifruit with H_2_S prolonged postharvest storage time, alleviated senescence, and prevented tissue softening (Gao et al., 2013). Furthermore, H_2_S treatment decreased MDA content, increased LOX activity, and reduced ROS production (Gao et al., 2013).

### H_2_S Action in Postharvest Physiology: The Non-climacteric Fruit Model

#### Strawberry

Strawberry (*Fragaria × ananassa* Duch.) is a non-climacteric fruit that is highly acceptable by consumers for its excellent sensory traits. However, strawberry fruit is highly prone to deterioration during harvest and storage as a result of its soft texture, while it is also susceptibility to fungal pathogens during postharvest period (Wills, 1996). A recent study revealed that H_2_S prolonged postharvest life of strawberry fruits (cv. Bao Jiao) according to the applied dosage (Hu et al., 2012). H_2_S fumigation on strawberry fruits retained higher content of reducing sugars, soluble proteins, free amino acids, and sustained flesh freshness and firmness. Additionally, H_2_S treatment kept respiration intensity and polygalacturonase (PG) activity at low levels. H_2_S modulated the antioxidant metabolism by increasing the enzymatic activity of CAT, POD, APX, and GR and lowering the activity of LOX and overall ROS levels, thus alleviating lipid peroxidation (Hu et al., 2012).

Zhang et al. (2014) reported a synergistic effect of H_2_S and NO toward the prolongation of postharvest life of strawberry fruits. Notably, the combination of H_2_S and NO in strawberry fruits suppressed fruit decay, inhibited the respiration rate, maintained crust color, and prolonged fruit firmness. The combination of H_2_S and NO also increased the enzymatic activity of chitinase (CHI), beta-1,3-glucanase (GNS) and decreased the activities of pectin methylestaerase (PME), PG, and endo-β-1,4-glucanase (EGase), consequently extending the shelf-life of strawberry fruits after harvest (Zhang et al., 2014), supporting the idea of a possible interplay between H_2_S and NO in postharvest life extension.

#### Mulberry Fruit

The production and consumption of mulberry fruit has witnessed a rapid increase during the past decade, due to recognized nutritional values and biological activities. However, fruits easily lose their postharvest commercial value due to rapid ripening rate. Hu H. et al. (2014) found that fumigation with H_2_S, released from 0.8 mm NaHS solution, enhanced the intercellular H_2_S content via the enhanced activity of D-cysteine desulfhydrase and L-cysteine desulfhydrase in mulberry fruits (*Morus indica* L. Dianmian-1). In addition, H_2_S delayed the decay of mulberry fruits, depressed respiration rate, and maintained quality characteristics (e.g., TA and ascorbic acid). Meanwhile, H_2_S fumigation exerted a protective role against senescence induced oxidative stress by terminating the propagation of lipid peroxidation and enhancing various antioxidant enzymes activity (Hu H. et al., 2014).

#### Grape

Grapes are subject to postharvest senescence during storage, in the syndromes of rachis browning, serious water loss, berry softening, off-flavor occurrence, as well as decay caused mainly by *Botrytis cinerea*, which reduces the commodity and consumption of grapes (Crisosto et al., 2001). The active role of H_2_S in postharvest senescence of grape berries (*Vitis vinifera* L × *Vitis labrusca* L, cv. Kyoho) was previously reported in the work of Ni et al. (2016). Exogenous application of H_2_S attenuated the rotting and threshing of grape berries. Prior to postharvest storage, fumigation of grape berries with H_2_S preserved in high levels several quality markers like firmness, soluble solids, TA, ascorbic acid, flavonoids, total phenolics, reducing sugars, and soluble proteins. As a result, the positive role of H_2_S in preserving chlorophyll and carotenoid content in both grape rachis and pulp was established. In the same work, H_2_S fumigation reduced the accumulation of ROS and MDA in grape pulp, while increased the activity of antioxidant enzymes and minimized LOX activity (Ni et al., 2016). Notably, the authors raised a question whether H_2_S might act as an antagonist to ethylene-induced fruit senescence.

## The Impact H_2_S on Postharvest Prolongation and Pathogen Inhibition

Solid evidence indicated that H_2_S contributes to the maintenance of postharvest shelf life via pathogen inhibition (Fu et al., 2014; Hu K.D. et al., 2014). It has been reported that endogenous H_2_S plays a crucial role in plant defense when agricultural crops suffer from fungal infections (Bloem et al., 2012). In the work of Fu et al. (2014), H_2_S exerted a positive antifungal effect against postharvest pathogen, namely, *A. niger* and *Penicillium italicum*, when they were inoculated on apples, kiwifruit, pear, sweet oranges, and mandarin. In the same study, H_2_S inhibited spore germination, germ tube elongation, and mycelial growth, produce abnormal mycelial contractions, and stimulate antioxidative enzyme activity in fruits. Similar results were also reported by Hu K.D. et al. (2014) where fumigation of pear fruits with H_2_S resulted in direct inhibition of the pathogens *A. niger* and *P. expansum*. The positive effect of H_2_S fumigation toward the inhibition of postharvest fungal attacks highlights the commercial importance of H_2_S.

## Possible Aspect of H_2_S Modes of Action in Postharvest Fruit Biology

The experimental evidence presented in this review suggested that H_2_S might influence fruit postharvest responses; however, it remains unclear how H_2_S could implement its anti-ripening effect. Recent evidence suggests that there is a putative interplay between H_2_S and ethylene (Ge et al., 2017). These authors demonstrated, as mentioned above, that H_2_S alleviated banana fruit ripening and expressed an antagonistic effect toward ethylene. In support, H_2_S fumigation at low concentration suppressed the expression of genes associated with ethylene biosynthesis under low ethylene environment in broccoli florets and apple slices (Li et al., 2014; Zheng et al., 2016), indicating that H_2_S function could be linked to ethylene perception. It has been recently proposed that H_2_S may bind to the copper ion of the same ethylene receptor as 1-MCP (Al Ubeed et al., 2018). However, the allosteric effect on the protein receptor would differ from coordination of H_2_S and 1-MCP (Pirrung et al., 2008), since several ethylene receptors are present in agricultural produce to which H_2_S may bind (Lacey and Binder, 2014). Even though H_2_S has an equal size to ethylene, it needs to be deprotonated so as to bind tightly to the receptor binding domain (Al Ubeed et al., 2018). Of relevance to postharvest physiology, the ability of H_2_S to bind to protein receptors was justified by Ge et al. (2017) where the parallel fumigation of H_2_S with ethylene increased the expression of ethylene receptor genes (*MaETR, MaERS1*, and *MaERS2*).

Aside from ethylene perception and signaling, evidence suggests that H_2_S can also function in autophagy. Analysis of the *Arabidopsis des1* mutant impaired in the cytosolic production of H_2_S from cysteine (Cys) led to the conclusion that H_2_S acts as an inhibitor of autophagy (Álvarez et al., 2012). Accordingly, postharvest fruit senescence associated with cell organelle-specific autophagy process could also be associated with H_2_S, as already evidenced in animals (Wang et al., 2012). H_2_S-mediated signaling in autophagy might be based on the reversible post-translational modification of the enzymes involved in the ubiquitylation process or of other proteins involved in the initiation and completion of the autophagosome (Batoko et al., 2017).

As discussed elsewhere (Hancock and Whiteman, 2016), it is likely that H_2_S will interact with ROS and NO through sulfate metabolism; for example, the presence of H_2_S can lead to an increase in reduced glutathione (GSH), thus affecting redox state homeostasis. This mechanism can have profound effects on the gene expression and the activity of proteins (as discussed further below). Another possibility is that H_2_S signaling may be mediated by *S*-nitrosothiol (SNO) metabolism and signaling of cells as well as enzymatic (e.g., nitric oxide synthase, nitrate reductase, xanthine oxidase)-dependent NO production. Furthermore, the nucleophilic properties of this molecule and its capacity to react with oxygen, H_2_O_2_, or peroxynitrite (ONOO^-^) suggest that it acts by reducing cellular oxidative stress (Nagy, 2015), which is commonly observed during fruit ripening (Molassiotis et al., 2013). This is also justified by the fact that postharvest treatments of H_2_S can counteract oxidative damage and stimulate the antioxidant enzymes activity in several fruits (Hu et al., 2012; Ni et al., 2016).

Among the many kinds of amino acid residues susceptible to oxidative stress, sulfur-containing amino acids, like methionine (Met) and cysteine (Cys), are the most sensitive (Møller and Sweetlove, 2010). Meanwhile, thiol groups in Cys residues are the main protein targets of *S*-nitrosylation (i.e., the covalent bonding of an NO moiety to Cys thiol side chain, to form SNO and the resulting S-nitrosoprotein; Lounifi et al., 2013). Recent studies also suggest that the key H_2_S signaling action is achieved by the modification of Cys residues to form a persulfide group (also known as persulfidation; Aroca et al., 2015). Thus, ROS, NO, and H_2_S competitively target Cys residues to exert their biological action, and therefore it is likely that a tight link between oxidation, nitrosylation, and persulfidation exists, that in turn may control oxidative- and nitrosative-based climacteric fruit ripening events. More importantly, if these thiols are being modified by H_2_S, then they are no longer accessible to be modified by ROS and NO, so the capacity for such signaling may well be severely altered in the presence of H_2_S (Hancock and Whiteman, 2016). This hypothesis is supported by results in citrus cells showing that conformational changes induced in specific proteins by *S*-nitrosylation could lock the structure of these proteins in a state under which they are no more sensitive to irreversible carbonylation induced by ROS (Tanou et al., 2009). Current evidence also suggests that protein *S*-sulfhydration adheres closely to the generally acknowledged paradigm for *S*-nitrosylation. Indeed, many of the protein sites reported to undergo endogenous *S*-nitrosylation have also been found to undergo *S*-sulfydration (Lu et al., 2013). These observations clearly indicate that it is essential to better understand the interplay between *S*-sulfhydration/desulfhydration and *S*-nitrosylation/denitrosylation in fruit biology.

In addition to having an influence on these specific redox-based protein modifications, it is likely that many H_2_S-driven signaling components and mechanisms involved in fruit metabolism have yet to be unraveled. For example, H_2_S may exert its mode of action *via* the modulation of energy metabolism related with the TCA cycle, glycolysis, electron transport chain, sustaining high levels of ATP which delay fruit senescence (Fotopoulos et al., 2010; Li et al., 2017). In this regard, it has been recently proposed that H_2_S can have two effects on mitochondrial electron transport chain activity. It can feed electrons into the pathway, with a concomitant increase in ATP production, or, alternately, it can inhibit complex IV, thus inhibiting ATP production (Hancock and Whiteman, 2016). Critically, it is significant to note that, the signal transduction pathway activated by exogenously applied H_2_S donors-produced H_2_S in various fruit systems experience postharvest handling may differ from the pathway induced by endogenously H_2_S generation, as previously suggested for the extracellular and intracellular NO signaling in stressed plant cells (Molassiotis et al., 2010).

## Conclusion and Perspectives

**FIGURE 1:**
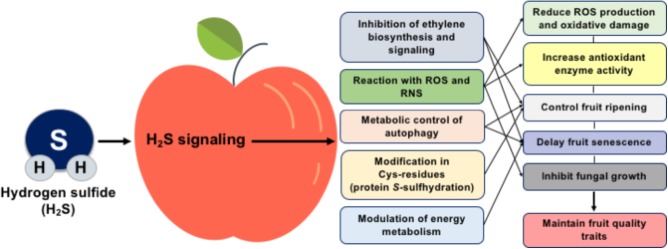
Schematic presentation of the mechanisms and potential benefits of H_2_S in postharvest fruit biology.

During the last decade, the manipulation of fruit postharvest loses is becoming a hot issue, and multiple lines of evidence discussed above propose an important role for H_2_S in postharvest fruit biology (**Figure 1**). In this sense, the exact role of H_2_S in fruit metabolism needs to be further characterized using high-throughput systems biology techniques such as transcriptomic, epigenomic, proteomic, and metabolomic approaches, while the synergistic effect of H_2_S with other molecules, such as ethylene and NO, should also be addressed. It is particularly significant to reveal how H_2_S could influence the ethylene perception mechanism and to characterize the functional significance of ethylene biosynthesis and response genes following H_2_S application through *in silico* studies at the genome-wide scale. Considering the importance of protein *S*-sulfhydration in various cellular responses, the regulatory system of post-translational modification of protein cysteine residues in fruit senescence must be elucidated. Although nitrosative and especially oxidative stress responses during fruit senescence are well studied, information regarding the interaction of H_2_S with nitro-oxidative postharvest conditions is scarce. Such understanding will lead to the establishment new technologies and strategies to preserve postharvest fruit quality and extent their postharvest life.

## Author Contributions

VZ initiated the project, collected and analyzed the data, and wrote the manuscript with input from all authors. AM provided feedback and reviewed the article. VF reviewed the article. GT devised the project, the main concepts ideas, and proof outline and reviewed the article. VZ, AM, VF, and GT conceived the idea, designed the structure of the text, and wrote the paper.

## Conflict of Interest Statement

The authors declare that the research was conducted in the absence of any commercial or financial relationships that could be construed as a potential conflict of interest.
